# Prediction of perioperative transfusions using an artificial neural network

**DOI:** 10.1371/journal.pone.0229450

**Published:** 2020-02-24

**Authors:** Steven Walczak, Vic Velanovich

**Affiliations:** 1 School of Information, Florida Center for Cybersecurity, University of South Florida, Tampa, FL, United States of America; 2 Department of Surgery, Morsani College of Medicine, University of South Florida, Tampa, FL, United States of America; Thomas Jefferson University, UNITED STATES

## Abstract

**Background:**

Accurate prediction of operative transfusions is essential for resource allocation and identifying patients at risk of postoperative adverse events. This research examines the efficacy of using artificial neural networks (ANNs) to predict transfusions for all inpatient operations.

**Methods:**

Over 1.6 million surgical cases over a two year period from the NSQIP-PUF database are used. Data from 2014 (750937 records) are used for model development and data from 2015 (885502 records) are used for model validation. ANN and regression models are developed to predict perioperative transfusions for surgical patients.

**Results:**

Various ANN models and logistic regression, using four variable sets, are compared. The best performing ANN models with respect to both sensitivity and area under the receiver operator characteristic curve outperformed all of the regression models (*p* < .001) and achieved a performance of 70–80% specificity with a corresponding 75–62% sensitivity.

**Conclusion:**

ANNs can predict >75% of the patients who will require transfusion and 70% of those who will not. Increasing specificity to 80% still enables a sensitivity of almost 67%. The unique contribution of this research is the utilization of a single ANN model to predict transfusions across a broad range of surgical procedures.

## Introduction

Surgical patients frequently require transfusions both during and immediately following surgery. Transfusions have been shown to improve patient outcomes and reduce mortality [1–3], as well as length of stay and readmission [4]. However, patients requiring transfusion are often also at greater risk for postoperative morbidities and mortality [5], such as: blood borne infections, ABO incompatibility, hemolytic reactions, transfusion-associated circulatory overload, transfusion related acute lung injury, immunosuppression [6], increased risk of perioperative infection [7], and earlier recurrence of malignancies after resection [8,9]. Preoperative knowledge of likely patient transfusion requirements would improve blood resource management, inform clinicians of the likelihood for postoperative morbidities, lower cost by avoiding preoperative typing and crossing patients unlikely to need a transfusion, and could serve to further improve patient outcomes [10].

The ability to identify patients who would be most likely to receive blood transfusions could greatly help in perioperative and postoperative planning and preparation. Regression models have been previously used to predict or model operative and postoperative transfusions [10,11]. Besides regression, artificial neural networks (ANNs) are also a reliable choice for modeling and problem solving in medicine [12].

ANNs are a machine learning methodology based on modeling the neuronal activity of the human brain and have been widely used across various disciplines of medicine [13,14]. Prior research indicates that ANNs generally outperform comparative regression models [12].

ANNs have been used in prior research to predict the surgically-related transfusion needs of patients, but they have all been for a single type of operation. Prior research has used ANNs to predict the transfusion needs of surgery patients undergoing abdominal aortic aneurysm repair [15] and coronary artery bypass grafting [16] operations. Other ANN-based transfusion prediction research is also singularly focused and includes transfusion needs for patients of trauma [17], and acute myeloblastic leukemia [18].

Our research goal is to evaluate the efficacy of using a single ANN model to preoperatively predict perioperative transfusion needs of patients over a wide spectrum of operations. This will significantly extend prior research that has developed ANN prediction models that only work for a single type of operation or disease. A corollary benefit will be the examination of several variables to determine their contribution to the ANN model’s prediction accuracy and subsequent explanatory power of the variables. Although the primary emphasis of this research is to prove that a single ANN may be used for transfusion prediction across any type of surgery, the examination of the impact of the variables selected is important to clinicians [19–21] and hence why two of the selected input variables for the ANN are further examined to establish their clinical contribution to a transfusion decision.

## Materials and methods

The study was approved by the institutional review board of the University of South Florida. The American College of Surgeons National Surgical Quality Improvement Program’s (NSQIP) participation use files (PUF) for the time period of 1/1/2014 to 12/31/2015 were used for the analysis. A total of 1,636,438 records of surgical procedures were obtained. The 2014 dataset was used for model development, while the 2015 dataset which was unique from the 2014 dataset was used for model validation. The validation data set was used only a single time for each version of the ANNs developed, emulating prospective use of the ANN on new out-of-sample data. Results are only reported for the 2015 validation dataset. Although retrospective, applying the ANN models trained using 2014 data on 2015 data for validation simulates the real-world ongoing generalization capabilities of the ANN models. For example, a new ANN model developed from a 2020 dataset using the method and variables described next, should have similar performance in predicting transfusion requirements of patients undergoing any type of surgery in the year 2021.

ANN models were developed using NeuralWorks^®^ Professional II/Plus to predict the intraoperative and/or postoperative blood transfusions for patients undergoing any type of surgery. Variables for the ANN transfusion prediction model were selected following review of 36 prior research articles on predicting or administering surgical transfusions and/or surgical bleeding. The prevalence of various variables used in these prior studies is shown in Table 1. Variables were selected using a heuristic of 12.5% prevalence in the literature examined, thus a variable is used in the ANN models if it was mentioned in at least 5 prior research articles which is a 12.9% prevalence. An exception is made for diabetes, which although the diabetes variable did not meet the initial inclusion heuristic, diabetes is mentioned as a bleeding risk factor in a sufficient number of other research publications that are not specific to surgery [55–60]. The variables selected following the prior research variable selection heuristic method (as shown in Table 1) were: age, sex, body mass index (BMI), presence of diabetes, hematocrit level, platelet count, international normalization ratio (INR), and creatinine.

**Table 1:** Values used in prior research to predict surgical transfusions or bleeding.

Factor	Number of times used	References
age (in years)	12	[15,16,18,22–30]
anemia	2	[26,31]
ASA or other score	4	[15,16,32,33]
blood pressure	3	[34–36]
BMI / weight	11	[16,22,24,27–30,32,37–39]
creatinine	5	[16,25–28]
diabetes	3	[26–28]
fibrinogen	1	[36]
heart rate	2	[34,35]
hematocrit	9	[15,28,29,31,32,35,37,40,41]
hemoglobin	17	[15,22,24–26,28,30,31,35,36,40,42–47]
history of heart disease	3	[15,16,29]
history of pulmonary disease	1	[15]
history of smoking	2*	[16,48]
LV-EF	4	[16,26,27,29]
platelets	9	[15,16,18,36,43,44,49–51]
PT / INR	7 / 3	[15,36,43,46,49,50,52]
PTT	4	[36,49,50,52]
sex	14**	[15,16,18, 22–24,26–29,37,45,53,54]
type of trauma	2	[34,36]
white blood cell count	1	[18]

* one study by Jung et al. showed smoking had no effect.

** one study by Millett et al., showed sex had no effect.

ANN variables should not be highly correlated [61,62] to avoid noise and overly strong output effects from the correlated variables, so a Pearson’s correlation matrix is used to validate that all the input variables are relatively uncorrelated, with the matrix shown in Table 2. Sex is a Boolean variable, diabetes is a categorical variable, and all other variables are continuous real values. Text data from the NSQIP database were converted to the appropriate type for the sex and diabetes variables. All data were normalized prior to training to prevent undue influence from variables that held much larger values than the rest of the variables: age, BMI, and platelet count.

**Table 2:** Input variable correlation matrix.

	Age	Sex	BMI	Diab. 1	Diab. 2	Hct	Platelet count	INR	Creat
Age	1								
Sex	-0.033	1							
BMI	-0.148	0.065	1						
Diabetes 1	0.054	-0.035	0.107	1					
Diabetes 2	0.110	-0.032	0.120	-0.107	1				
Hct	-0.131	-0.209	0.101	-0.156	-0.039	1			
Platelet count	-0.142	0.175	0.013	0.009	-0.010	-0.109	1		
INR	0.087	-0.061	-0.009	0.058	0.007	-0.147	-0.026	1	
creatinine	0.077	-0.148	0.005	0.186	0.009	-0.187	-0.075	0.093	1

All matrix values rounded to 3 decimal places.

In addition to these variables used in the transfusion prediction models, presence of bleeding disorders, co-morbidity severity as measured by the American Association of Anesthesiologists (ASA) classification, whether the operation was elective or an emergency, operation complexity as measured by the work relative value units (w RVU), operation time (in minutes), and mortality were recorded as demographic data. The demographic data is used to determine similarities and differences between transfused and non-transfused data, but again was not used as input to the ANN models.

**Fig 1:**
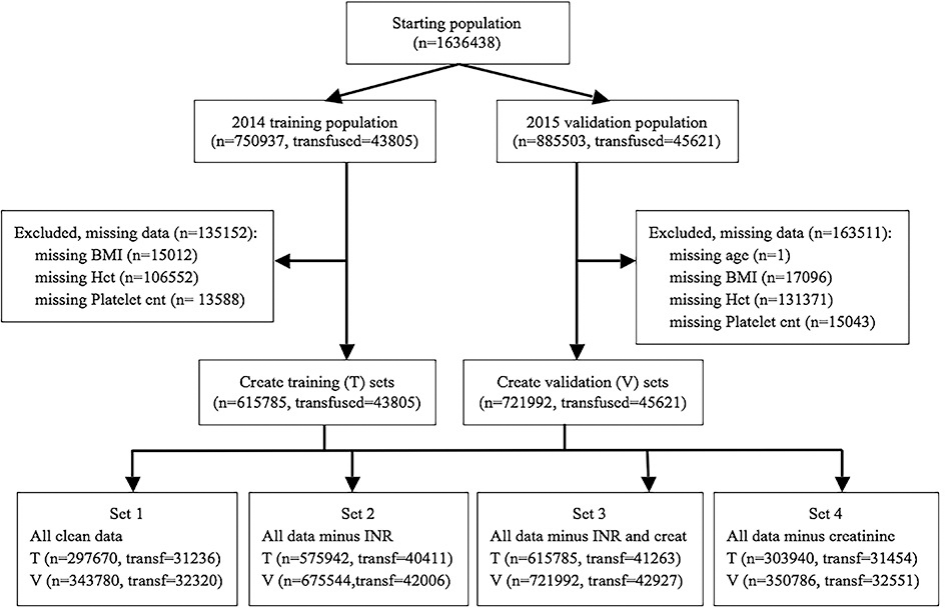
Cohort selection flow diagram.

Records for individual patient operations were eliminated from use in both the training and validation datasets if they were missing any of the ANN variable values other than INR or creatinine, with the cohort flow diagram shown in Fig 1. The output or dependent variable is the number of units transfused of either whole blood or packed red blood cells, since these are the only values reported for transfusions in the NSQIP database. The goal of the ANN output is to predict whether or not a transfusion will be needed by a specific surgery patient, thus any non-zero dependent value is used to indicate a transfusion will occur, while zero output values indicate that no transfusion will be needed by the patient.

Four different ANN transfusion and regression transfusion models are developed around different combinations of the heuristically selected variables, dependent on the presence or absence of INR and/or creatinine. Set 1 uses all of the variables listed: age, sex, BMI, diabetes, hematocrit, platelet count, INR, and creatinine. Set 2 use the same variable set as Set 1, but eliminates the creatinine variable. Set 3 uses the same variable set as Set 2, but also eliminates the INR variable. Set 4 uses the same variables set as Set 1, but eliminates just the INR variable. As shown in Fig 1, the number of training and validation samples for each model ranged from 297670 training samples and 343780 validation samples for Set 1 with all variables present to 615785 training samples and 721992 validation samples for Set 3, which had the fewest number of variables missing both INR and creatinine.

Supervised learning techniques of backpropagation (BP) and radial basis function (RBF) were both attempted along using varying quantities of hidden nodes per layer and different numbers of hidden layers (one or two), following recognized ANN research methodology [61–63]. BP training is selected due to its very wide usage in clinical decision support models so as to facilitate comparison of results with other ANN clinical decision making models [13,64]. RBF training is selected as an alternative training method due to its proven performance when extrapolation is required [65].

**Fig 2:**
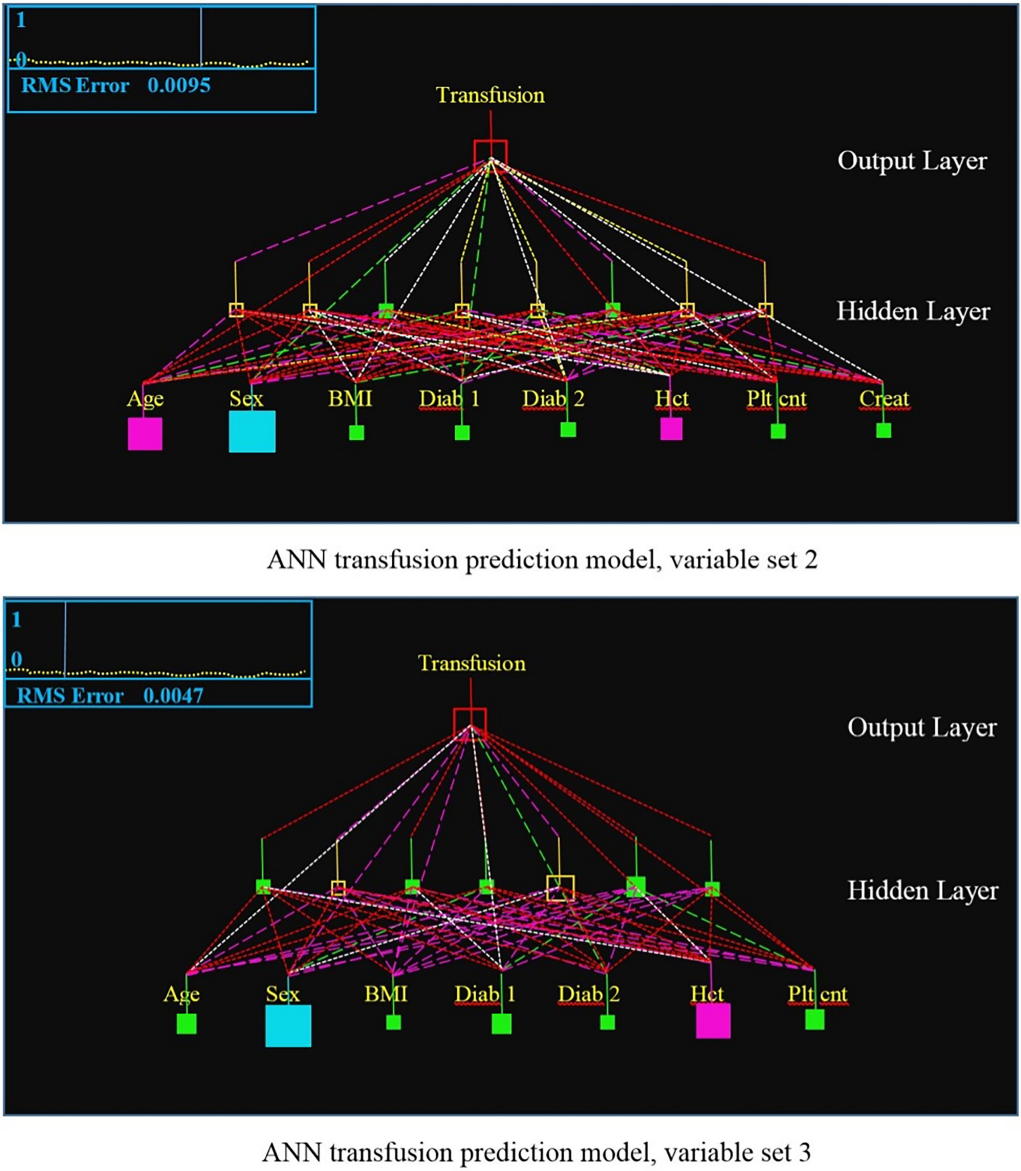
Sample ANN architectures for variable sets Set 2 and Set 3. Size of the boxes representing the processing elements in the ANN indicates the size of numeric difference from zero. Hollow boxes indicate negative values and filled in boxes indicate zero or positive values. Dashed lines represent the weighted connections between processing elements, with the length of the dashes indicating positive or negative values and the color indicating relative value.

The initial architecture for each ANN model used a hidden layer composed of a number of nodes equal to the input layer, or preceding hidden layer in the case of the two hidden layer backpropagation trained model. Training occurred for one million training epochs and also required that the root-mean-square error (RMSE) for training was below 0.01, with an epoch size of 6 samples. If after one million training epochs the RMSE was still not below 0.01, then training continued using 1000 epoch training size iterations until the desired RMSE was reached. The reason that just a RMSE stopping condition is not used to stop training is due to the very large number of non-transfused patients, which caused the ANN training RMSE to go below 0.02 and sometimes 0.01 within 20–30 thousand epochs, but resulted in poor performance on the validation set due to the fact that this used one half to less than one third of the possible training samples to be evaluated by the ANN during training. The number of hidden layer nodes was then increased or decreased by 2, separately for each hidden layer, until no further improvement in performance as measured by sensitivity for a 70% specificity was detected for the validation set. Interestingly, this happened very quickly for the BP models. The best performing architecture for variable Sets 2 and 3 is a single hidden layer model with the hidden layer nodes equal to the number of input nodes. The best performing ANN architecture for variable Sets 1 and 4, containing the INR variable, is a two-hidden layer BP trained model with the first hidden layer equal to two times the number of input nodes minus 1 and the second hidden layer being half the size of the first hidden layer. All of the connection weights were always randomized anytime one of the ANN models was trained. An example of the backpropagation trained ANN model architectures for transfusion prediction with a single hidden layer for Sets 2 and 3 are shown in Fig 2. Each validation set is used only a single time for each model following training.

Examining the contribution of using ANN modeling for transfusion predictions is facilitated by comparing the ANN results against a comparable logistic regression model, since regression is one of the most popular clinical decision making tools [12,66]. Because a binary decision is being modeled: whether or not a patient will require a transfusion, logistic regression is the appropriate choice for the type of regression. Regression models for each of the four data sets are developed using MatLab^®^.

Because both the ANN models and the logistic regression models produce a continuous value, a single cutoff value is need for each model to determine which values, those above the cutoff, represent a required transfusion (sensitivity) and which values below the cutoff represent no need for a transfusion (specificity). While it is possible to maximize sensitivity to 100% with a very small cutoff value, this also reduces the specificity to the point where a transfusion is predicted for almost all patients. Therefore, the heuristic of maximizing sensitivity while at the same time having a specificity of 70–80% is used to select cutoff values for both the ANN and regression models. The cutoff values, which started at zero, were determined using the training data output were modified by one tenths and then by one hundredths and could also be altered by 5 thousandths to move the respective specificity level towards either 70% or 80%, with the closest value at or above the respective heuristic specificity value chosen using these cutoff value guidelines. Respective model performance is determined using the level of sensitivity achieved for specific pre-defined levels of specificity on the out-of-sample validation set from surgeries performed during 2015.

### Statistical methods

The nonparametric machine learning method of backpropagation trained ANNs is the primary statistical method utilized for development of the transfusion prediction models, as described above. Logistic regression models are also developed for each of the four variable sets evaluated with the ANN models.

Area under the receiver operator characteristic (AUROC) curve for each of the ANN and regression models is calculated using trapezoidal approximation. Statistical significance between the differences of the various models and AUROC values are calculated using a Chi-squared test for differences, since this test is nonparametric and does not require a normal distribution of either the data or the variances [67]. The Chi-square tests for differences are run at a 0.05 significance level with one degree of freedom.

## Results

The surgical patient population demographics for the largest sets of training and validation data, Set 3, which is a superset of all other data sets, are shown in Table 3. There are no clinically significant differences between the 2014 model development cohort and 2015 model validation cohort. However, there were important clinically significant differences between the transfused and non-transfused patients for both the 2014 model development and 2015 model validation cohorts. Transfused patients were, on average, older, more likely to have diabetes mellitus and bleeding disorders, have more severe co-morbidities (as assessed by ASA class), more likely to undergo emergency operations, which were more complex (as measured by wRVU’s), and requiring longer time to complete. These patients also had higher creatinine values, INR values and PTT values. Lastly, transfused patients had a significant increase (*p* < .01 using a standardized t-test with known variances) in the rate of 30-day mortality compared to non-transfused patients with transfused patients being 14 times more likely to die.

**Table 3:** Surgical patient demographics.

Factor	2014 Training Set		2015 Test Set	
	Transfused (n = 41,254)	Not Transfused (n = 709,682)	Transfused (n = 45,621)	Not Transfused (n = 839,881)
Sex (% Male: Female)	42.8: 57.2	43.0: 57.0	42.8: 57.2	43.4: 56.6
Age, years (mean ± standard deviation, range)	65.4±14.6 (18–90+)	55.8±16.4 (18–90+)	65.0±14.7 (18–90+)	55.6±16.5 (18–90+)
Height, in. (mean ± standard deviation, range)	65.3±4.2 (41–82)	66.0±4.1 (40–84)	65.4±4.3 (42–91)	66.±4.1 (40–95)
Weight, lbs. (mean ± standard deviation	172.0±50.0 (53–580)	186.6±50.7 (37–657)	172.3±50.5 (56–667)	188.2±51.3 (44–882)
ASA Distribution (%)	I: 1.24	I: 10.33	I: 1.16	I: 9.41
	II: 18.05	II: 46.93	II: 17.24	II 46.39
	III: 53.33	III: 37.90	III: 54.33	III: 39.21
	IV: 25.74	IV: 4.76	IV: 25.40	IV: 4.90
	V: 1.71	V: 0.0008	V: 1.87	V: 0.0009
Diabetes (%)	24.0	14.6	24.1	15.0
Bleeding Disorders (%)	12.9	3.5	14.1	3.7
INR (mean ± standard deviation, range)	1.16±0.38 (0.1–10)	1.07±0.29 (0.1–10)	1.16±0.40 (0.1–10)	1.07±0.29 (0.1–10)
PTT, sec (mean ± standard deviation, range)	32.3±10.4 (6.7–120)	30.4±7.2 (6.2–120)	32.5±11.3 (5.2–120)	30.3±7.4 (5–120)
Hematocrit, % (mean ± standard deviation, range)	33.7±6.4 (8.1–59)	39.8±4.9 (8–60)	33.6±6.6 (8–60)	40.1±5.0 (8–60)
Platelet Count, 1,000/mm^3^ (mean ± standard deviation, range)	252.2±115.3 (1–992)	249.0±77.6 (1–998)	254.4±116.8 (1–1,000)	249.1±78.2 (1–1,000)
Creatinine	1.25±1.23 (0.1–14.72)	0.99±0.85 (0.1–15)	1.26±1.27 (0.1–15)	0.99±0.84 (0.1–15)
Elective Surgery (%)	52.9	80.8	51.0	80.7
Operative Time, mins. (mean ± standard deviations, range)	191.7±145.6 (1–1314)	100.9±82.9 (1–1440)	200.3±149.4 (1–1440)	104.8±84.9 (1–1435)
wRVU (mean ± standard deviations, range)	24.45±10.81 (0.33–92.99)	15.87±8.26 (0.3–83.12)	24.53±10.89 (0.3–83.12)	16.20±8.51 (0.3–108.91)
Mortality (%)	5.6	0.4	5.7	0.4

The backpropagation learning algorithm produced better performing ANN models than the RBF, with respect to maximizing sensitivity at the specified specificity levels. The sensitivity, specificity, and AUROC curve values for the backpropagation trained ANN models and the logistic regression models are reported in Table 4. Recall that the best performing architectures for variable sets 1 and 4 are both two hidden layer architectures and the best performing architecture for variable sets 2 and 3 are both single hidden layer architectures. Significance values in Table 4 are calculated using Chi-square at a .05 significance level. From Table 4, the Set 2 ANN had the best overall sensitivity performance at both the 70% specificity and 80% specificity levels. However the Set 3 ANN had the best AUROC values, indicating superior performance for all levels of desired specificity. The ANNs for data sets 2 and 3 both outperformed all of the regression models, however the regression models did perform better on data sets 1 and 4 over the corresponding ANN models. The best AUROC value for the ANN model using variable set 3 is statistically significantly better than all other models except for the ANN model for variable set 2 for which there was not significance difference. Specifically, the ANN model using variable set 3 was *p* < .01 better than the regressions models for variable sets 1 and 4 and *p* < .001 better than all of the other models, based on the Chi-squared test for differences.

**Table 4:** ANN and regression model results for each of the 4 data sets.

Data Set	Prediction model	Sensitivity	Specificity	AUROC
**Set 1**:	ANN	71.8%	70.1%	
	Training^a^	62.2%	80.0%	
	ANN	73.7%	70.0%	0.814
	validation	64.5%	80.0%	
	Regression	74.2%	70.3%	0.832
		64.3%	80.8%	
	ANN	75.7%	70.1%	
	Training^a^	65.8%	80.1%	
**Set 2**:	ANN validation	75.6%*	70.6%	0.847**
		66.6%**	80.6%	
	Regression	73.7%	70.7%	0.816
		64.1%	80.7%	
**Set 3**:	ANN Training^a^	73.1%	70.0%	
		62.6%	80.1%	
	ANN validation	75.4%*	70.1%	0.858**
		66.2%**	80.0%	
	Regression	72.9%	71.6%	0.815
		64.2%	80.5%	
**Set 4**:	ANN Training^a^	75.7%	70.1%	
		65.0%	80.1%	
	ANN validation	73.7%	70.0%	0.832
		64.4%	80.1%	
	Regression	74.1%	70.5%	0.831
		64.0%^†^	81.0%	

^a^ Training set values are provided at the request of a *PLoS One* reviewer. AUROC values are not supplied for the training set, since these will never be used in practice.

* *p* < .05, ANN model better than regression model.

** *p* < .01, ANN model better than regression model.

A total of 2678 different current procedural terminology (CPT) codes, which are used to report different surgical procedures, are represented in the ANN training and validation data sets. These codes came from 531 different hospitals across the United States in 2014 and 615 hospitals in 2015. The operations represented by these CPT codes are shown in Table 5. The training set had 2498 CPT codes, of which 155 were not present in the validation set. The validation set had 2523 CPT codes of which 180 were not present in the training set. The large number of diverse CPT codes will help validate the research goal of demonstrating the efficacy and generalization of a single ANN model for predicting transfusions for patients of any operation, not just a single operation as has been done in prior research.

**Table 5:** Surgeries performed for 2014 training and 2015 validation data sets.

Operation type	Number performed 2014	Number performed 2015 (transfusions)	2015 Sensitivity^b^ at 70% Specificity
**Cardiac Surgery**			
coronary arterial	1858	2152 (1193)	59.9%
intrathoracic great vessel	739	786 (320)	62.2%
pericardium / non-coronary	1455	1473 (776)	59.4%
**ENT Surgery** **(Ear, Nose, Throat)**^a^	6150	7121 (181)	75.7%
**General Surgery**			
adrenal	857	931 (57)	61.4%
anorectal	7598	8087 (625)	71.5%
biliary	40008	47009 (585)	83.1%
breast	26108	28567 (213)	55.9%
colon	84396	97411 (6365)	83.9%
esophagus	6480	7076 (344)	79.1%
non-ENT head/neck	14893	16599 (46)	71.7%
hernia repair	52947	60627 (389)	71.2%
laparotomy	7494	8661 (681)	80.3%
liver	4919	5842 (961)	62.5%
lymphatic	2327	2641 (90)	65.6%
omentum	172	160 (11)	72.7%
pancreas	7316	8002 (1500)	75.3%
skin / soft tissue	295	354 (13)	100%
small bowel	8138	8841 (1002)	85.7%
spleen	807	904 (261)	90.4%
stomach	26524	28385 (920)	72.2%
**Neurosurgery**			
cranial / brain	8110	9618 (488)	58.6%
peripheral nerve	393	551 (2)	100%
spine	19834	24136 (870)	56.8%
**Obstetrics / Gynecology** **Surgery**			
female genitalia	926	1093 (3)	100%
ovarian/fallopian tube	4396	5263 (729)	68.6%
uterus / cervix	38854	46197 (1891)	58.5%
vaginal	6506	7155 (44)	54.5%
**Orthopedic Surgery**			
arthroscopy	12557	15458 (13)	92.3%
bone / muscle / extremity	287	398 (4)	100%
hand	4435	5724 (27)	81.5%
hip / pelvis	38042	49626 (7412)	84.0%
lower extremity	51145	67198 (3782)	80.9%
shoulder	4848	6133 (168)	80.4%
spine	18894	22579 (1638)	47.4%
upper extremity	1896	2538 (97)	93.8%
**Plastic Surgery**			
bone graft	17	21 (4)	50.0%
breast	9620	10245 (183)	25.7%
head/neck	8	2 (0)	N/A
omentum	31	32 (3)	100%
skin / soft tissue	9885	11874 (838)	86.8%
**Thoracic Surgery**			
chest wall	491	537 (25)	88.0%
diaphragmatic	135	148 (12)	41.7%
mediastinal	887	883 (22)	63.6%
tracheal / lung	7974	8385 (522)	82.4%
**Urology Surgery**			
bladder	7787	9318 (923)	82.9%
kidney	7392	9055 (823)	71.8%
male genitalia	2406	3025 (24)	91.7%
prostate / urethra	14707	18471 (438)	60.0%
ureter	334	403 (38)	73.7%
**Vascular Surgery**			
endovascular	5331	5589 (659)	81.6%
intra-thoracic great vessel	0	1 (0)	N/A
non-aortic arterial	32416	34823 (3604)	79.2%
aortic	1224	1249 (861)	57.4%
embolectomy / thrombectomy	1270	1301 (231)	74.0%
vein	1266	1334 (16)	93.8%
**Total all surgery types**	615785	721992 (42927)	

^a^ ENT operations include: larynx, glossectomy/tongue, mandible, palate, ENT tumor excision, and other ENT head and neck operations.

^b^ Sensitivity is specified for the ANN using variable Set 3, which had the highest AUROC value, at a 70% specificity level.

Table 5 also reports the number of operations for each type of surgery in the 2015 validation set that had transfusions and also the ANN variable Set 3 sensitivity at a 70% specificity level for each operation type, which yielded the 75.4% overall sensitivity value for the ANN. Only 2 specific types of operation, out of the 56 types reported, had no transfusion and this was for a total of 3 operations out of the 721992 operations predicted by the ANN model. This demonstrates that although the transfusion prevalence may be low for some types of operations, transfusions do occur across a wide variety of operation types, which further demonstrates the need for transfusion prediction models capable of working across a large collection of surgery types. Remembering that all of the transfusion predictions made for all of the surgeries reported in the NSQIP-PUF dataset were made by a single ANN prediction model, the Set 3 validation sensitivity does vary across the different types of operation reported, but only 3 out of the 56 operation types, representing 4.3% of the operations for which a transfusion is administered, were below 50% sensitivity.

## Discussion

Preoperative assessment of patients at risk for intraoperative and postoperative hemorrhage and need for blood transfusions is both a patient safety issue and a cost issue. Obviously, in cases of life-threatening intraoperative hemorrhage, blood transfusions are potentially lifesaving. However, “routine” type and cross-matching of blood is a costly and potentially wasteful endeavor. Each type and cross-match Medicare payment is $37.30 with an additional $17.96 for each additional unit cross-matched [68]. The ability to accurately classify 70–80% of patients who will not require any transfusion, along with identifying a corresponding 66–75% of patients who will need a potentially lifesaving transfusion, can reduce surgical blood costs by eliminating a large number of unneeded type and cross-matches for those patients identified as not requiring a transfusion for operations that would typically perform a type and cross-match.

The standard approach to assessing operative bleeding risk includes history, physical examination, laboratory evaluation (INR, PTT, platelet count, bleeding time), type of operation, patient co-morbidities (e.g., inherited coagulopathies, coagulopathy of liver disease), drug-induced coagulopathy (e.g., anticoagulation therapy for cardiovascular disease), and platelet dysfunction (e.g., immune thrombocytopenic purpura) [6,69]. Our patient data has also shown that age, ASA class, presence of diabetes mellitus and bleeding disorders were more associated with patients receiving transfusions. Operations on or near large vessels, those with extensive dissections, and prolonged operations are generally considered heuristics of a higher risk of bleeding and consequent need for transfusion. Our data do reflect that patients receiving transfusions were more likely to undergo more complex operations (as measured by w RVU values) and longer operations. Although useful, these heuristics have not been precise in identifying the amount a patient will bleed or who will need a transfusion [70,71]. Therefore, better predicting models would be useful and ANNs provide the opportunity to create better transfusion prediction models.

In addition to being a machine learning method able to learn solutions to arbitrarily complex problems [72], ANNs have several other advantages over regression and other statistical methods that serve to improve medical model prediction performance, including: they are nonparametric thus data is not required to fit specific prerequisite requirements and therefore surgeons do not have to have advanced statistical analysis training [72], all variable interactions do not need to be predefined since they are discovered through machine learning, and once trained they are highly resistant to noise or errors in the data for the input variables. This has been demonstrated in the current research by the fact that 180 new operations (CPT codes) were included in the results for the ANN that were not present in the training set, thus these operations were completely new to the ANN prediction model.

The principle drawback of ANN models in medicine is their black box nature [72] and subsequent difficulty in determining which input variables are most significant for clinical decision making regarding surgical transfusions. Several techniques exist for trying to determine the explanatory power of input variables, such as iteratively leaving out select variables [72,73] or summing of the connected weights [74]. The leave-one-out strategy is used in the current research to measure if INR or creatinine had any purpose or explanatory power in transfusion prediction modeling. The results displayed in Table 4 show that the data sets that removed the INR variable performed better than the other data sets, which indicates that this variable is not contributing to the determination of a future transfusion need for any specific patient. The lack of affect from the INR variable might be caused by high correlation with other variables included in the input variable set and as such acts as noise to the ANN, reducing performance [62]. A Pearson’s correlation matrix is calculated for all variables in the data set (see Table 2) and no unusually high correlations were detected, with the highest absolute correlation value being 0.209 indicating a small and acceptable level of correlation and validating the finding that INR, which had a maximum absolute correlation value of 0.147, does not contribute to transfusion predictions when used with the other indicated variables. This is in contrast to the findings from Table 3, which indicate that transfused patients would have a higher INR, however the ANN demonstrates this value is unnecessary for predicting transfusions. The fact that two hidden layer architectures performed better than single hidden layer architectures for the ANN transfusion prediction models that utilize the INR variable (variable Sets 1 and 4), indicates that the solution surface when INR is considered has additional nonlinearities [61]. Future research could examine why the introduction of an INR value creates a more complex solution surface for predicting transfusions.

The data set that removed just creatinine (Set 4) from the full set of variables (Set 1) had similar performance, with no statistically measurable difference to the full data set. The data set that removed creatinine and INR both did show improved performance and had the highest AUROC value. Future research is needed to further investigate the importance of creatinine and other different variable combinations, where variables are selected to minimize correlation and to maximize predictive performance. Identification of contributing and non-contributing variables has clinical significance in that contributing variables are further identified as key elements in the transfusion process. Non-contributing variables may successfully be removed from the decision making process, making the transfusion decision more efficient by reducing potential noise from non-contributing variables and less costly by eliminating the need to perform specimen collection and the subsequent pathology to acquire these unneeded values.

ANNs should definitely be in any clinical medicine researcher’s toolbox, as demonstrated by the results for variable sets 2 and 3. However, from Table 4, depending on the specific variables used, regression modeling may perform as well as or slightly better than ANNs. Thus, clinical researchers should utilize both ANNs as well as regression and possibly other machine learning techniques such as random forests and then compare the results of all models to select the best performing model and consequently optimize clinical outcomes.

It is interesting to note from Table 3 that postoperative deaths within 30 days of surgery occurred 14 times more frequently in patients who required transfusions. The non-transfused mortality rate was 4 per 1000 patients, while the transfused mortality rate was 56–57 per 1000 patients. The rates of postoperative morbidity were also significantly higher in transfused patients. Higher mortality and morbidity rates for transfused patients may be due to the difficulty of the operation, but the transfusions themselves may play a role [6]. The majority of the complications in the transfused patients are infectious, thus the immunosuppressive nature of transfusion may affect postoperative morbidity. The fact that transfused patients are much more likely to suffer mortality or postoperative morbidity indicates that preoperative knowledge of the likelihood for a transfusion to be needed perioperatively, could also be used by anaesthesiologists and surgeons to proactively plan treatment and antibiotic regimens for these patients in addition to improving the management of blood supply.

Bleeding and transfusion prediction models have been developed in a number of specialties, including trauma [75], orthopedic surgery [76,77], cardiac surgery [78,79], vascular surgery [80], liver transplantation [81], and otolaryngology [82]. Our study is unique in that rather than assessing transfusion need for a single type of operation, we can do it across a broad spectrum of operation types. Most prior transfusion estimation models relied on conventional statistical techniques, such as multiple logistic regression modeling. However, our studies demonstrates that the ANN modeling methodology can be superior to logistic regression when the best independent variable set is used. The ANN model using variable Set 2 accurately predicted the transfusion needs for two thirds of the surgical patients who would need one and for over 80% of those who would not require any transfusion and the ANN model using variable set 3 produced a significantly higher AUROC. If a larger false positive rate can be tolerated at a 70% specificity level, then the ANN model is able to identify over three fourths of patients for any type of surgery who will need a transfusion, assuming the pre-operative labs would include either hematocrit or hemoglobin and also platelet count. The false positive rate identifying individuals as needing a transfusion who ultimately did not receive one could have a negative effect on the blood supply, but does not negatively affect the clinical outcomes for these patients, other than a heightened vigilance for morbidity that is less likely to occur.

Others have used ANN to predict bleeding due to medical or surgical therapy. These include lower gastrointestinal hemorrhage [83], cardiopulmonary bypass [84], experimental hemorrhage model in rats [85], and radiation therapy for prostate cancer [86]. Although none of these studies examined consequent transfusion requirements. Hayn, et al. [87] used predictive modeling based on decision trees on a large dataset of multiple different types of operations to predict transfusion needs. These efforts reflect the need to better predict blood loss and consequent transfusion requirements.

### Limitations and future research

There are limitations to the reported ANN transfusion prediction model. Firstly, as intraoperative hemorrhage is related to surgical trauma and surgeon skill, reducing blood loss and transfusion need is very much operator dependent. In addition, although length of operation and extent of dissection can be anticipated by the operating surgeon to a certain extent, these factors are still quite variable. There is no obvious way to include this in a preoperative prediction model. Restrictive blood transfusion practices [88] were also not able to be represented in the variables for the ANN model. Future research is needed to examine if analogous values for the actual length and difficulty of an operation may be used in the transfusion prediction ANN model and how this will affect performance of the ANN models.

Although 2678 different operations in total were evaluated, with 2523 different operations in the validation set, these are all operations that reported the values for the variables used in the ANN models. Any operation that did not record a hematocrit value, a platelet count value, age sex, BMI, or diabetes status would not have been included in either the training or validation sets for the ANN models. While the ANN demonstrated very good sensitivity for the operations evaluated, its performance on the operations that did not report one of the required variable values is undetermined. However, the lack of a hematocrit value specifically may indicate that the operation being performed had an extremely low probability of requiring a transfusion and as such it is generally safe to not evaluate transfusion requirements for these operations.

Additionally, the current ANN models predict perioperative transfusion need for up to one year following the training date. As long as there are no new surgery protocols, procedures, or technologies that impact patient bleeding and transfusions, then the current model should be able to continue predicting transfusions at the documented sensitivity and specificity levels until such change occurs. Therefore, retraining of the ANN may be unnecessary until an advancement in surgical protocol, process or technology occurs. Future research should examine how long diagnostic and prognostic ANN models may be used before re-training becomes necessary, based on decreased sensitivity and specificity values.

As mentioned, the NSQIP database used for the reported research only reports transfusion of whole blood or packed red blood cells. Other types of blood products may also be transfused, such as plasma or platelets. The platelet count variable was not removed from any of the ANN models attempting to determine the impact of variables because it is considered the primary indicator for platelet transfusions and the ANN model is designed to work for any type of transfusion. Since platelet transfusions were not reported in the NSQIP database, the validity of the model for predicting platelet transfusions in addition to whole blood and packed red blood cell transfusions could not be ascertained. Future research is needed to attain databases of surgical procedures that also report the transfusion of platelets in addition to other blood products and use this data to further evaluate the efficacy of the proposed ANN model for preoperative transfusion predictions.

Finally, since not all possible ANN architectures were evaluated for each of the backpropagation and RBF trained models, this represents another possible limitation in that a slightly different architecture could possibly improve the performance of the ANN surgical transfusion prediction models. Thus, the reported results should be interpreted as the minimum possible sensitivity achievable for the defined specificity levels and consequent AUROC values. Future research in addition to examining different variable combinations to further clarify the impact of each variable could also examine the various architectures not evaluated by the current research, specifically incrementing or decrementing the number of hidden nodes in each layer by one instead of two.

### Clinical implications

The research reported in this article demonstrates the efficacy of using a single ANN model to predict the transfusion requirements of patients across a wide range of operations as shown in Table 5. This leads to the question of how the ANN results can be used in clinical practice. The NeuralWorks^®^ Professional II/Plus shell tool used to develop the ANN transfusion prediction models comes with a feature that enables the ANN to be produced in an executable format, utilizing the C programing language, which may be run from a command line prompt. Clinicians should not be expected to know how to execute the ANN executable application and thus a user interface would be required to be produced that would either automatically acquire the requisite input variable values or prompt the clinician for missing values. The user interface would then execute the encoded ANN and translate the results into a more readable format, such as reporting a “high likelihood for a transfusion,” when the ANN predicts a transfusion occurring.

Automatic acquisition of variables could be accomplished by linking the ANN model with existing electronic medical record (EMR) systems or surgical planning systems, such as One Medical Passport^®^ (see https://www.onemedicalpassport.com/). Prior research has shown that clinical decision support systems like ANNs may be connected and embedded with EMR systems [89]. Because of the documented improvements in clinical decision making when clinicians utilize EMR systems [90], integrating the ANN transfusion prediction decision support system with an EMR or surgical planning system would facilitate access by integrating the ANN transfusion prediction decision support tool into their workflow, which would further improve blood bank planning and surgical and post-operative preparation [91] and consequently improve patient outcomes. The ANN would be able to alert surgeons to possible unexpected complications that would lead to a transfusion even if none were anticipated and could also be used to confirm the necessity of surgeon blood orders. Ultimately though, the decision to type and cross-match and ultimately transfuse blood products is the surgeon’s and the ANN-based clinical decision support system should be used as evidence to assist surgeons in making those decisions.

## Conclusion

ANNs have been shown to efficaciously provide a mechanism to preoperatively evaluate the potential for need of perioperative transfusions for individual patients across a wide range of operation types. A single ANN model has been shown to work at 66–76% sensitivity with a corresponding 80–70% specificity, with a corresponding AUROC of 0.847 to 0.858, across a very large number of different operations (2678 CPT codes). This advances the state-of-the-art where in the past ANN models have only predicted transfusions for a single type of operation. Additionally, the sensitivity and specificity performance is across data from 615 different hospitals indicating good generalization of these results for any hospital choosing to use this ANN-based preoperative transfusion model. Incorporation of other anticipated factors, such as difficulty and length of the operation, may additionally increase its predictive value.
